# Lung tumour growth kinetics in SPC-c-Raf-1-BB transgenic mice assessed by longitudinal in-vivo micro-CT quantification

**DOI:** 10.1186/1756-9966-31-15

**Published:** 2012-02-20

**Authors:** Thomas Rodt, Christian von Falck, Sabine Dettmer, Katja Hueper, Roman Halter, Ludwig Hoy, Matthias Luepke, Juergen Borlak, Frank Wacker

**Affiliations:** 1 Dept. of Diagnostic and Interventional Radiology, Hannover Medical School, Carl-Neuberg-Str. 1, 30625 Hannover, Germany; 2 Dept. of Pharmaceutical Research und Medical Biotechnology, Fraunhofer-Institute for Toxicology and Experimental Medicine, Nikolai-Fuchs-Str. 1, 30625 Hannover, Germany; 3 Institute of Biometry, Hannover Medical School, Carl-Neuberg-Str. 1, 30625 Hannover, Germany; 4 General Radiology and Medical Physics, University of Veterinary Medicine Hannover, Bischofsholer Damm 15, 30173 Hannover, Germany; 5 Institute for Pharmaco- and Toxicogenomics, Hannover Medical School, Carl-Neuberg-Str. 1, 30625 Hannover, Germany

**Keywords:** Micro-CT, lung tumour, transgenic mouse model, growth kinetics

## Abstract

**Background:**

SPC-c-Raf-1-BxB transgenic mice develop genetically induced disseminated lung adenocarcinoma allowing examination of carcinogenesis and evaluation of novel treatment strategies. We report on assessment of lung tumour growth kinetics using a semiautomated region growing segmentation algorithm.

**Methods:**

156 non contrast-enhanced respiratory gated micro-CT of the lungs were obtained in 12 SPC-raf transgenic (n = 9) and normal (n = 3) mice at different time points. Region-growing segmentation of the aerated lung areas was obtained as an inverse surrogate for tumour burden. Time course of segmentation volumes was assessed to demonstrate the potential of the method for follow-up studies.

**Results:**

Micro-CT allowed assessment of tumour growth kinetics and semiautomated region growing enabled quantitative analysis. Significant changes of the segmented lung volumes over time could be shown (*p *= 0.009). Significant group differences could be detected between transgenic and normal animals for time points 8 to 13 months (*p *= 0.043), when marked tumour progression occurred.

**Conclusion:**

The presented region-growing segmentation algorithm allows in-vivo quantification of multifocal lung adenocarcinoma in SPC-raf transgenic mice. This enables the assessment of tumour load and progress for the study of carcinogenesis and the evaluation of novel treatment strategies.

## Background

A number of genetic animal models of lung cancer has been developed to better understand the molecular causes of this disease. In-vivo imaging in these disease models can allow a better understanding of biological processes and a time-course assessment of therapeutic approaches. We here report on longitudinal in-vivo micro-CT measurements of lung tumour in a transgenic mouse model of lung cancer.

The animal model examined has been reported in the literature already [1-5]. In the SPC-c-Raf-1-BB (referred to as SPC-raf) transgenic mouse model overexpression of the serine-threonine-kinase c-raf to alveolar epithelium is achieved by use of the surfactant protein C (SPC) promoter. Raf is an essential constituent of the mitogen activated protein kinase (MAPK) signalling pathway, that has been found to communicate a cell surface receptor signal to the DNA in the nucleus [4]. This MAPK pathway is often found to be dysregulated in human malignancies [3]. Essentially, the targeted overexpression in SPC-raf transgenic animals results in adenocarcinomas of the lung, with multifocal adenomatous hyperplasia being defined as the earliest proliferative lesion of dysplastic cells. Histopathology of this animal model has been obtained at different time-points to show the time course of the tumour progression. The first distinct morphological changes seen by histopathology have been reported to occur by the age of 2 months. By the age of 8 month, approximately 60-70% of the lungs have been reported to be tumour, as judged by histopathology. At the age of 12 months advanced tumour stage can be found macroscopically, affecting the entire lung [3].

This animal model allows probing for mechanisms of carcinogenesis based on a genetic cascade that also plays a crucial role in the development of adenocarcinoma of the lungs in humans. Furthermore, it offers the opportunity to study carcinogenesis in a more realistic setting as compared to models of implanted (xenograft) tumours into immunodeficient mice. In fact, the animals are still immunologically competent, while the continuous expression of the transgene secures continuous tumour pressure. Thus, the relevance of overexpressed protooncogenes or disabled tumour suppressor genes can be studied.

Different imaging modalities have been reported and their advantages and disadvantages have been evaluated for imaging of murine lung pathology. Comparatively fast assessment of morphology can be obtained using micro-CT [6]. Furthermore, metabolic information on the examined tissue can be provided by the use of other modalities such as micro-positron emission tomography (PET), magnetic resonance imaging (MRI) or optical imaging [7-9]. Spatial correlation with morphological information, e.g. by micro-PET/micro-CT registration, allows precise localization of this information on metabolism. More recently, molecular imaging of responsiveness to chemotherapy at the tumour site or imaging of disease candidate genes has been reported.

In this study we report on the use of a micro-CT quantification algorithm for the longitudinal assessment of tumor progression in SPC-raf transgenic mice.

## Methods

### Animals

12 mice (SPC-raf transgenic n = 9 and wildtype n = 3) were examined (Table 1). Transgenic mice were maintained as hemizygotes in the C57 BL/6 mouse strain background, polymerase chain reaction was used to secure transgenic status. All experiments were performed according to a protocol as approved by the local regulatory authorities (No. 33-42502-06/1081, Lower Saxony State Office for Consumer Protection and Food Safety, Germany).

**Table 1:** Animals examined in this study

**Animal No**.	Genetical status	Sex	Follow-up (d)	Thoracic organs (g)	Body weight (g)	Thoracic organs/body weight
1	SPC-raf	F	399	1.49	23.03	0.05

2	SPC-raf	F	362	1.22	18.70	0.07

3	SPC-raf	M	536	1.44	36.95	0.04

4	SPC-raf	F	466	1.34	23.63	0.06

5	SPC-raf	F	466	1.02	17.90	0.06

6	SPC-raf	F	466	0.95	17.78	0.05

7	SPC-raf	M	547	1.44	28.77	0.05

8	SPC-raf	M	546	1.15	29.93	0.04

9	wild-type	M	547	0.49	50.20	0.01

10	wild-type	M	546	0.45	47.00	0.01

11	wild-type	M	398	-	-	-

12	SPC-raf	F	146	-	-	-

Sex and age at last micro-CT are given. Note that female animals have shorter follow-up times (see discussion). In animals 11 and 12 no histology was obtained. The ratio of thoracic organ weight and total body weight clearly shows the differences between transgenic and control animals at necropsy.

In-vivo micro-CT imaging was first performed at the age of 2 months (day 55 to day 61) and repeated every 4 weeks. Follow-up examinations were repetitively carried out until the animal had to be euthanized due to medical condition or termination of the study. The follow-up had to be terminated on day 146 in one animal, in the other animals between day 362 and 547. A total of 156 CT exams were carried out in this study.

Isoflurane inhalation anaesthesia was administered using a nose cone. The animals were placed in prone position on a multimodality bed that enables changes between the different imaging modalities without repositioning. A pressure transducer pad was placed under the animal's chest for respiratory monitoring, which was used for respiratory gating and for control of anaesthesia.

### Micro-CT

Non contrast-enhanced prospectively respiratory gated micro-CT was performed (GE Explore Locus, General Electric Healthcare, Chalfont St. Giles, UK) with an effective pixel size of 0.094 mm (80 kV, 450 μA, 360 projections/scan, exposure time/projection 100 ms, scan technique 200°, 4 × 4 detector bin mode). The scan FOV was 32.8 mm. For respiratory gating the signal from the transducer pad was used to generate the image acquisition time points using the software Biovet (m2 m Imaging, Newark, NJ, USA). Images of the chest were reconstructed and calibrated to the Hounsfield scale. Expected mean radiation dose was calculated to be 197 mGy based on phantom and cadaver measurements in a previous study [10].

### Histology

The imaging findings were correlated to necropsy and histology in 10 cases (8 transgenic and 2 control, see table1) by direct visual comparison. In two animals no histology was obtained. At necropsy lung surface was assessed for tumour affection and correlated to imaging. After necropsy the excised lungs were filled with Tissue-Tek O.C.T.^® ^(Sakura, Finetek Europe, NL) and subsequently fixed in 4% buffered formalin (pH 7.2). After dehydration (Shandon Hypercenter, XP) lungs were embedded in paraffin. Sections (2 μm thick) were deparaffinized with xylene and H&E stained.

### Post-Processing

**Figure 1:**
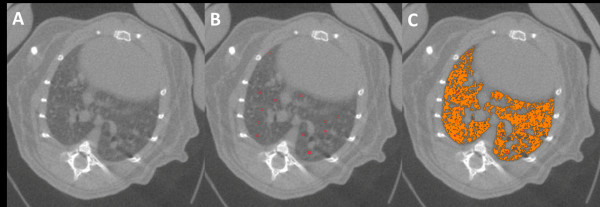
**Segmentation of aerated lung volume as a surrogate parameter to assess the multifocal tumor spread**. Here in control animal no intrapulmonary masses are seen in micro-CT imaging (**A**). Seed points are placed manually in the aerated lung (**B**). Segmentation of the aerated lung is performed by applying a region growing algorithm (**C**). The entire aerated parts of the lung are segmented. No spread of segmentation volume into adjacent structures occurred.

**Figure 2:**
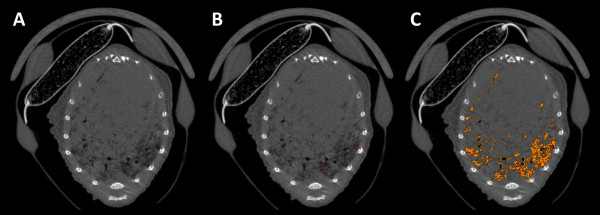
**Segmentation of aerated lung volume as a surrogate to assess the multifocal tumor spread in SPC-raf transgenic animal**. Micro-CT showing the distinctive diffuse bilateral tumour growth (A). Seed points are placed manually in the aerated lung (B). Segmentation of the aerated lung is performed applying a region growing algorithm (C). Note that the lung areas consolidated by tumour are correctly excluded from the segmentation volume, no overspilling of segmentation volume into adjacent anatomical structures.

For quantification of the multifocal tumours a segmentation of the aerated parts of the lungs was used as a surrogate parameter, as direct measurement was not feasible. A region-growing algorithm for micro-CT quantification of tumour load and progress for diffuse lung adenocarcinoma was established and validated earlier [11]. The open-source software MevisLab (Fraunhofer Mevis, Bremen, Germany) was applied, 20-40 seed points were used to generate the region growing segmentation with a segmentation threshold tolerance of 2% (Figure 1 and 2). For each data set 3 separate segmentations were performed and the results of the 3 measurements were averaged.

### Statistical analysis

Statistical analysis was performed using IBM SPSS Statistics 19 (IBM Corp., Armonk, NY, USA). A repeated measurement analysis was performed. Due to the limited number of animals the number of time points analysed had to be reduced. Analysis was performed for time points 2, 4, 6, 7-13 months. Due to a limited number of measurements one animal had to be excluded from the statistical analysis (see above, the animal had to be euthanized on day 146). Furthermore a linear regression analysis was performed and the correlation coefficient was calculated. *P *< 0.05 was considered as statistical significant.

## Results

### Micro-CT and Post-Processing

No adverse events occurred due to the imaging procedures or anesthesia. Image quality was good in most cases and acceptable in all cases.

**Figure 3:**
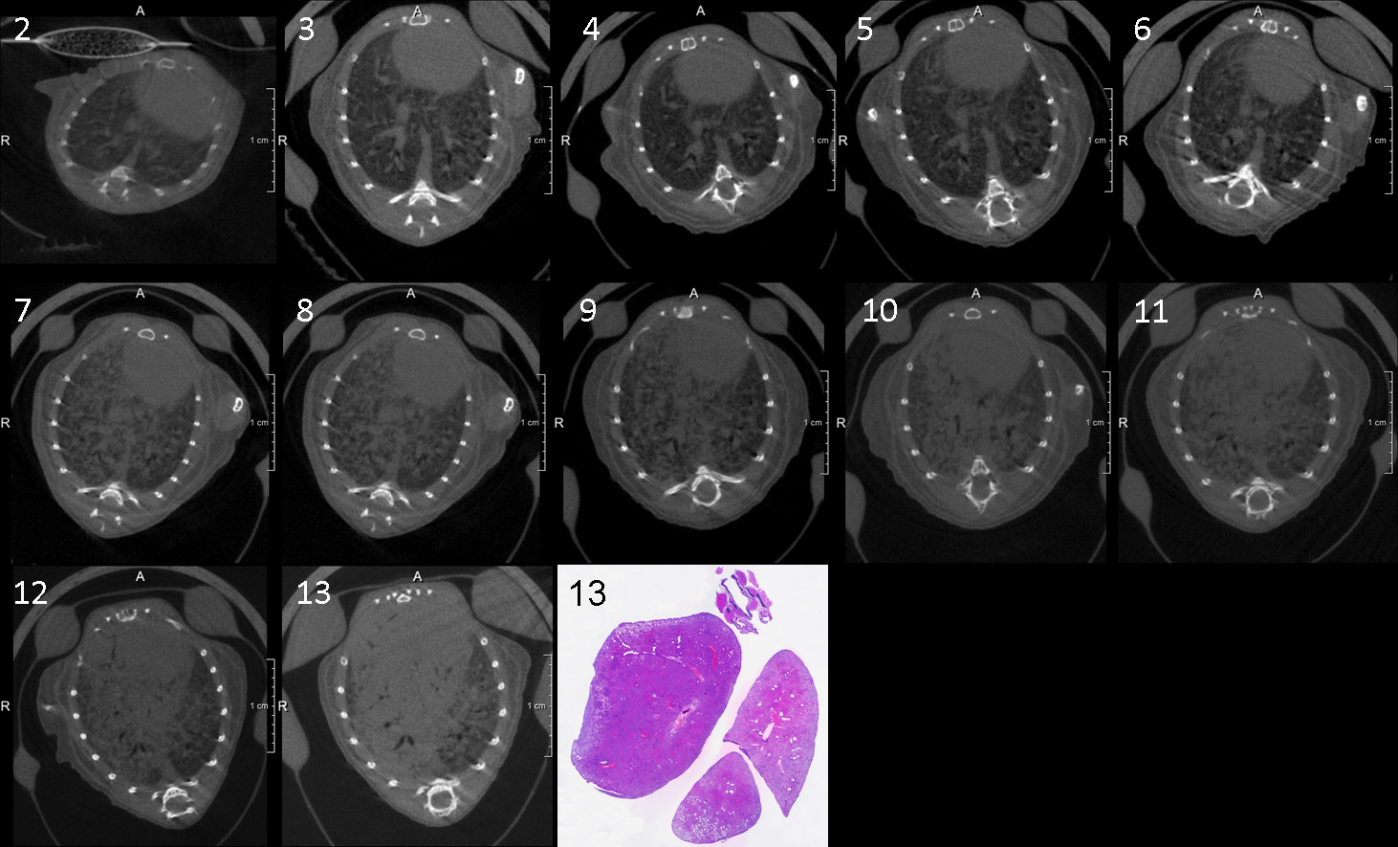
**Time-course of tumour progressing in micro-CT of a single SPC-raf transgenic animal (No.2; months 2-13)**. Axial slice orientation in corresponding positions. The multifocal tumour progression is clearly depicted. Histology at 13 months shows distinctive tumour burden in corresponding areas.

**Figure 4:**
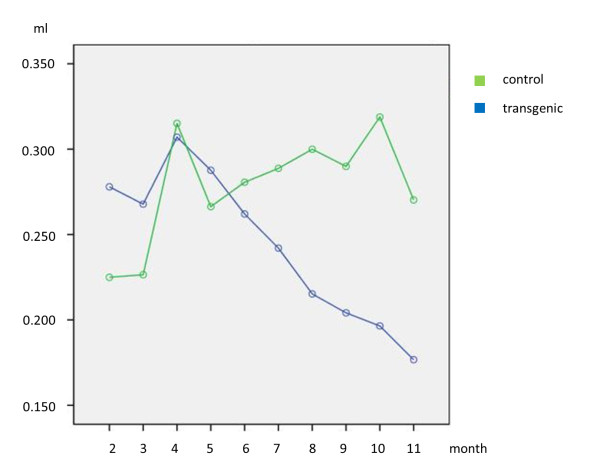
**Estimated marginal means of the segmentation volumes of the aerated parts of the lungs as an inverse surrogate parameter for tumour burden in SPC-raf transgenic (blue) and control animals (green) against time**. Initial increase is assumed to result from normal growth of the animals. Note the distinct separation of the curves from 5 months on. Statistical analysis of later timepoints showed significant differences (*p *= 0.043).

In this follow-up study progressive tumour burden could be seen in SPC-raf transgenic mice, while no obvious changes were noted in the control group (Figure 3 and 4). Visual correlation of histology and micro-CT at the corresponding time-point showed good accordance.

The region growing segmentation using the described post-processing algorithm could be performed in all cases. In the evaluation study repositioning of seed points due to overspilling of the segmentation volume into adjacent structures was not necessary using the same algorithm for 90 segmentations [11], in this study in repositioning was necessary in about 10 cases out of 468 segmentations. This necessitated reevaluation of the position of the chosen seed points and repositioning into aerated parts of the lungs. This way tumour burden and growth was assessed quantitatively using the decrease in aerated lung volume as a surrogate. The initial increase in lung volume in the first 4 months was attributed to normal growth.

In the comparatively small group examined here, tumour growth seemed to occur at a later point of time in male animals as compared to female animals. Female animals showed clinical signs of tumour necessitating sacrifice earlier compared to male animals.

### Statistical analysis

Repeated measurement analysis of the time points 2, 4, 6, 7-13 months showed significant changes of the segmented lung volumes over time (*p *= 0.009). Interaction of the measurements was rejected (*p *= 0.035). Testing for group differences did not show significant results, due to the small number of animals and the spread of lung volume at early time points in normal animals. Analysis of time points 8 to 13 months, when tumour progression occurs, showed significant group differences (*p *= 0.043). Linear regression analysis yielded equation 1 to calculate lung volume. The correlation coefficient was determined as R = 0.538.

(1) $$\text{Vo}{\text{l}}_{\text{lung}}=-0.01\times \left(\text{month}-1\right)+0.315\;\text{ml}$$

## Discussion

In this study we examined the tumour growth kinetics of SPC-raf transgenic mice by serial micro-CT examinations. Small animal imaging allows assessment using each animal as its own control in follow-up examinations. Given the relevant inter-individual spread it has the potential to optimize studies. To prevent intra-individual spread sophisticated imaging and post-processing techniques have to be established as elaborated below. An advantage of imaging especially in diffuse or multifocal pathologies is that the entire volume can be assessed additional to circumscribed areas of sectional histopathology obtained.

Very few studies on follow-up micro-CT examination have been performed in transgenic murine models of lung cancer (mainly K-ras transgenic) [12-14]. Other groups performed follow-up examination in single lesions caused by intrapulmonary injection of tumour cells or several/multiple lesions initiated by intraperitoneal injection of urethane [15-17]. To the best of our knowledge, no report on micro-CT assessment of tumour growth kinetics in the SPC-raf transgenic lung tumour mouse model has been published so far. Furthermore, the follow-up exams reported did usually include only a limited number of imaging time points as compared to up to 15 time points in this study, allowing a more detailed assessment of growth kinetics. Further studies have shown the use of micro-CT for the detection of primary lung tumours or pulmonary metastases without a follow-up being performed [7,18].

All the various imaging approaches of murine animal models of human lung tumour have different advantages and disadvantages. Some animal models are highly artificial with regard to the mechanism of carcinogenesis or the immunological competence of the tumour bearing organism. But they may provide imaging characteristics similar to the situation in humans with a single comparatively well-defined lesion. The multifocal tumour growth in the SPC-raf transgenic animal model examined in this study limits direct application of established radiological imaging techniques in humans. However, it has already been reported that this animal model allows examination of the potential relevance of human protooncogenes or disabled tumour suppressor genes in an immunologically competent environment. Thus, aspects of human carcinogenesis may be better understood highlighting the clinically translational aspects of the research.

In the animals examined in this study tumour growth seemed to occur at a later point of time in male animals as compared to female animals, furthermore females showed clinical signs of tumour necessitating to sacrifice the animals earlier compared to male animals (Table1). This has not been reported for the SPC-raf transgenic animal model, yet. However, due to the genetic mechanism of tumour induction in this model it might represent a relevant finding to understand lung tumour carcinogenesis. Further experiments have to be performed in order to validate the potential finding and present a hypothesis for the origin. The primary focus of this study was to demonstrate the use of micro-CT for assessment of tumour load and growth. The issue demonstrates the potential additional benefit of the method for assessment of cofactors affecting carcinogenesis applying intraindividual time-course assessment.

Micro-CT imaging applying the setup described above did not result in adverse events, even though animals had advanced tumour stages at the later time points. In synopsis with other studies performed we attribute this to the use of isoflurane inhalation anaesthesia, respiratory monitoring and the use of a heating blanket. We performed prospective respiratory gating as it has been reported to significantly increase image quality. However, more sophisticated gating techniques such as retrospective or intrinsic gating or high-speed single-breath hold techniques could further optimize imaging [19-22]. MRI has been reported to allow high spatial resolution imaging of the lung. Martiniova et al. even reported better detection of small lesions with MRI as compared to micro-CT [7]. Optical imaging techniques or micro-PET enable assessment of functional parameters but have limitations in high resolution imaging of morphology [8,23]. Various post-processing strategies have been reported allowing precise evaluation of specific aspects of the image data.

Dose measurements for the applied micro-CT protocol were performed in a phantom and ex-vivo in previous studies. The effective dose calculated from these measurements was 154 mGy for the selected protocol. An additional radiation dose has to be added for planning the scan-area under fluoroscopy. For this a dose of 19 mGy/min was measured, resulting in 202 mGy/scan [11]. Animals received between 4 and 15 repetitive exams with 4 weeks interscan interval (MV = 13.0, SD = 3.05). The calculated accumulative dose ranged from 808 mGy within 91 days (4 exams) to 3030 mGy within 475 d (15 exams). The mean calculated accumulative dose was 2626 mGy within approximately 450 d. These dose values in synopsis with a reported LD_50/30 _(dose that is lethal in 50% of the animals within 30 days) of 7.52 Gy demonstrate the relevance of the issue [24]. However, we consider direct adverse effects (structural changes to the lungs or unintended radiation effects on the tumour growth) to be unlikely. Although gene expression changes have been seen in cell cultures with doses as low as 20-500 mGy [25] structural changes like fibrosis were not even seen following doses as high as 7-9 Gy [24] and the reported values for therapeutic radiation also amounted to values as high as 15.5 Gy [12].

In conclusion the presented region-growing segmentation algorithm allows longitudinal in-vivo quantification of multifocal lung adenocarcinoma in SPC-raf transgenic mice. This enables the assessment of tumor load and growth kinetics for the study of carcinogenesis and the evaluation of novel treatment strategies.

## Competing interests

There are no financial or non-financial competing interests to declare in relation to this manuscript by any of authors.

## Authors' contributions

TR designed the study, contributed to performing the experiments and wrote the manuscript. CvF, SD, and RH participated in acquisition of the imaging data and contributed to drafting the manuscript. ML performed radiation dose analysis, furthermore he was involved in drafting the manuscript. LH performed statistical analysis and was involved in drafting the manuscript. JB and FW contributed to study design, data analysis and revised the manuscript critically. All authors read and approved the final manuscript.
